# Variation in the Microbiome, Trichothecenes, and Aflatoxins in Stored Wheat Grains in Wuhan, China

**DOI:** 10.3390/toxins10050171

**Published:** 2018-04-24

**Authors:** Qing-Song Yuan, Peng Yang, Ai-Bo Wu, Dong-Yun Zuo, Wei-Jie He, Mao-Wei Guo, Tao Huang, He-Ping Li, Yu-Cai Liao

**Affiliations:** 1 Molecular Biotechnology Laboratory of Triticeae Crops, Huazhong Agricultural University, Wuhan 430070, China; yqs198609031006@126.com (Q.-S.Y.); pp19870517@163.com (P.Y.); zuodongyun@webmail.hzau.edu.cn (D.-Y.Z.); heweijie@webmail.hzau.edu.cn (W.-J.H.); guomaowei@126.com (M.-W.G.); huangtao@mail.hzau.edu.cn (T.H.); hepingli@mail.hzau.edu.cn (H.-P.L.); 2 College of Plant Science and Technology, Huazhong Agricultural University, Wuhan 430070, China; 3 Key Laboratory of Food Safety Research Institute for Nutritional Sciences, Shanghai Institutes for Biological Sciences, Chinese Academy of Sciences, Shanghai 200031, China; abwu@sibs.ac.cn; 4 College of Life Science and Technology, Huazhong Agricultural University, Wuhan 430070, China

**Keywords:** deoxynivalenol, fungal diversity, *Fusarium graminearum*, metagenomics, nivalenol, wheat storage

## Abstract

Contamination by fungal and bacterial species and their metabolites can affect grain quality and health of wheat consumers. In this study, sequence analyses of conserved DNA regions of fungi and bacteria combined with determination of trichothecenes and aflatoxins revealed the microbiome and mycotoxins of wheat from different silo positions (top, middle, and bottom) and storage times (3, 6, 9, and 12 months). The fungal community in wheat on the first day of storage (T_0_) included 105 classified species (81 genera) and 41 unclassified species. Four species had over 10% of the relative abundance: *Alternaria alternata* (12%), *Filobasidium floriforme* (27%), *Fusarium graminearum* (12%), and *Wallemia sebi* (12%). Fungal diversity and relative abundance of *Fusarium* in wheat from top silo positions were significantly lower than at other silo positions during storage. Nivalenol and deoxynivalenol in wheat were 13–34% higher in all positions at 3 months compared to T_0_, and mycotoxins in wheat from middle and bottom positions at 6 to 12 months were 24–57% higher than at T_0_. The relative abundance of toxigenic *Aspergillus* and aflatoxins were low at T_0_ and during storage. This study provides information on implementation and design of fungus and mycotoxin management strategies as well as prediction models.

## 1. Introduction

Wheat is a staple food crop in many parts of the world, and has a per capita consumption of 78.46 kg/years in China. Annual global wheat production has increased steadily in recent years, reaching 733.8 million tonnes (MT) in 2015 after increasing from 713.2 MT in 2013 [1]. This has led to a rapid increase of the ending stocks from 189.4 MT in 2013 to 225.8 MT in 2015 [1]. China is the second largest producer of wheat in the world, and its annual production was 121.9 MT in 2013 and 130.2 MT in 2015. Accordingly, wheat stocks increased from 32.43 MT in 2013 to 40.23 MT in 2015 [1], about 40% of which is usually stored in farmers’ houses under natural conditions at room temperature with no ventilation facility. During storage, wheat often experiences mold growth and mycotoxin contamination, resulting in food safety concerns and huge losses [2].

Various fungal species have been found in stored wheat in different countries, ruining grains and resulting in unusable products [3,4,5]; mycotoxigenic fungi often cause the most severe damage to wheat. For instance, *Fusarium graminearum* was reported to be the most frequent species that contaminated durum and soft wheat in a central Italian area in 2009 and 2010, whereas *F. avenaceum* and *F. poae* only accounted for a small proportion of contamination [3]. In Iranian silos, wheat samples imported from Argentina contained six fungi, including toxigenic fungi *Fusarium* spp. and *Penicillium* spp. *Penicillium* spp., together with five other fungal species, were present in Australian wheat in which no *Fusarium* spp. were detected. In Kazakhstan, wheat grains that were stored in Karaj silos or unloaded from trains contained ten fungal genera, including the toxigenic species *Aspergillus* spp., *Fusarium* spp., and *Penicillium* [5]. In Saudi Arabia, wheat grains were contaminated by six fungal genera, and the most common toxigenic genera were *Fusarium* (29.1%), *Aspergillus* (14.3%), and *Penicillium* (9.3%) [4].

Non-mycotoxigenic fungi have also been identified in stored wheat grains. For instance, in Iranian silos, wheat grains contain *Rhizopus nigricans*, *Ulocladium* spp., *Cladosporium* spp., and *Alternaria* spp. In Kazakistan, *Mucor mucedo*, *Curvularia triticola*, *Ulocladium clamydosporium, Alternaria* spp. and *Cytospora tritici* [5] were identified. In Saudi Arabia, the most common fungal genera in stored wheat include *Alternaria* (8.2%).

Mycotoxins that contaminate wheat grains pose a serious and direct threat to the health of humans and domestic animals due to their high toxicity [6,7,8]. Fungal species from *Aspergillus*, *Fusarium*, and *Penicillium* are toxigenic producers that generate the most important mycotoxins and reduce quality of products [9,10,11,12,13,14,15,16]. In particular, *F. graminearum* and *A. flavus* produce widely distributed mycotoxins such as deoxynivalenol (DON), nivalenol (NIV), and aflatoxins [17,18]. In Iran, 10 of 34 wheat samples collected from three silos in Golestan province contained aflatoxins, with levels ranging from 0.23 ng/g to 7.08 ng/g [19]. In China, DON is the predominant mycotoxin that contaminates wheat, and 89% of harvested wheat grains from Anhui and Jiangsu provinces in the middle and downstream regions of the Yangtze river have been reported to contain DON, with levels from 993 to 2691 μg/kg [20]. Studies have also reported detection of aflatoxins in wheat flour, whereas the aflatoxin-producing fungi were not revealed [21,22].

In the present study, barcoded Illumina paired-end sequencing (BIPES) and chemical analyses were used to investigate the microbiome and mycotoxin profiles of wheat at different times and silo positions during one year of storage. The results showed that *F. graminearum* was one of the predominant toxigenic species, and DON was the predominant *Fusarium* mycotoxin, with substantial variations among storage times and silo positions. A relatively low abundance of *A. flavus* was detected, with a low level of aflatoxins. These findings are likely to encourage the implementation and design of mold and mycotoxin management strategies, as well as prediction models under storage conditions.

## 2. Results

### 2.1. Data Characteristics

Total DNA from all 39 wheat samples stored in three silos (three repeats; three positions in each silo, top, middle, and bottom) was isolated and used for PCR with a fungal ITS2 primer pair and a bacterial V3V4 primer pair. PCR results showed that all samples contained one clear fungal DNA fragment of about 500 bp in length and one bacterial DNA fragment of about 500 bp in length, as expected (Figure S1). These results indicated that all wheat samples contained both fungal and bacterial communities, and thus, the isolated DNA was subjected to BIPES.

Based on metagenomic sequencing of fungal ITS2 in stored wheat grains, the average number of raw paired reads generated per sample was 33,762. After filtering and de-noising, the average number of clean, paired reads was 29,409 (Table S1). Tags were assembled from paired reads and the average number of tags was 26,324 (Table S1). Lengths of tags ranged from 308 to 371 bp. These tags were subsequently clustered into different operational taxonomic units (OTUs), and the average number of OTUs was 205 (range: 20–466).

Bacterial V3V4 sequencing revealed that the average number of raw paired reads generated per sample was 33,046. After filtering and de-noising, the average number of clean, paired reads was 30,535 (Table S2). Tags were assembled from paired reads, and the average number of tags was 30,169. The lengths of all tags obtained were longer than 425 bp (Table S2). The tags were subsequently clustered into different OTUs, and the average number of OTUs was 117 (range: 38–239). The data were further used for variation analyses at different taxonomic levels across storage time and silo position.

### 2.2. Fungus Variation at the Phylum Level across Storage Time and Silo Position

**Figure 1:**
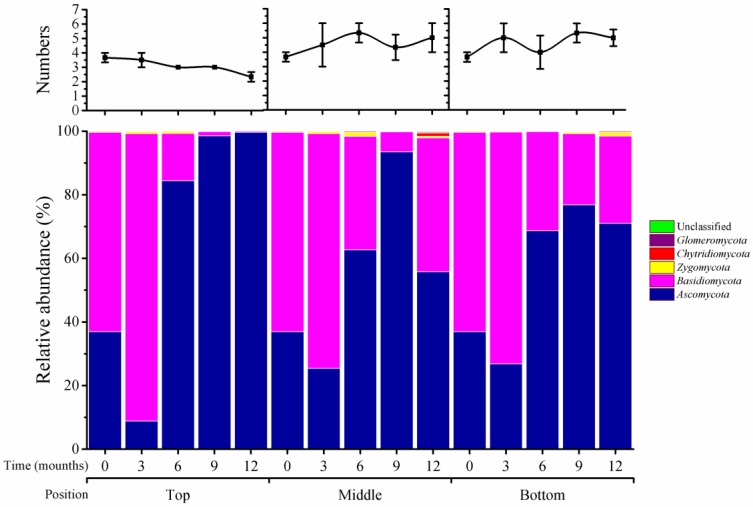
Distribution of fungi at the phylum level in wheat stored for 0–12 months at different silo positions. Top, middle, and bottom indicate silo positions. The numbers 0, 3, 6, 9, and 12 represent the storage times in months in silos. The top panel represents the variation in number of phyla, the values represent the means of three replicates with the standard deviation (SD); the bottom panel represents the distribution of fungi at the phylum level.

As shown in Figure 1, five fungal phyla were classified in stored wheat samples, including Ascomycota, Basidiomycota, Chytridiomycota, Glomeromycota, and Zygomycota, and their numbers varied across storage times and positions of silos. Unclassified fungi accounted for a small portion. At the top position of silos, there were four phyla at 0 and 3 months, three phyla at 6 and 9 months, and two phyla at 12 months. However, at the middle and bottom positions, phylum numbers increased from four at time 0 to five at 3 months and onward. The relative abundance of phyla also varied at different times and positions. For instance, the relative abundance of Ascomycota increased from 38% at time 0 to 99.7% at 12 months in top positions, while it reached 94% and 77% in the middle and bottom positions, respectively, at 12 months. Basidiomycota was at 55% at time 0, but had disappeared from the top position at 12 months; this phylum accounted for 48% and 22% at 12 months in the middle and bottom positions, respectively. Thus, storage time and position in silos had varied impacts on the type and abundance of fungi during wheat storage.

### 2.3. Variation in Fungal Genera across Storage Time and Silo Position

**Figure 2:**
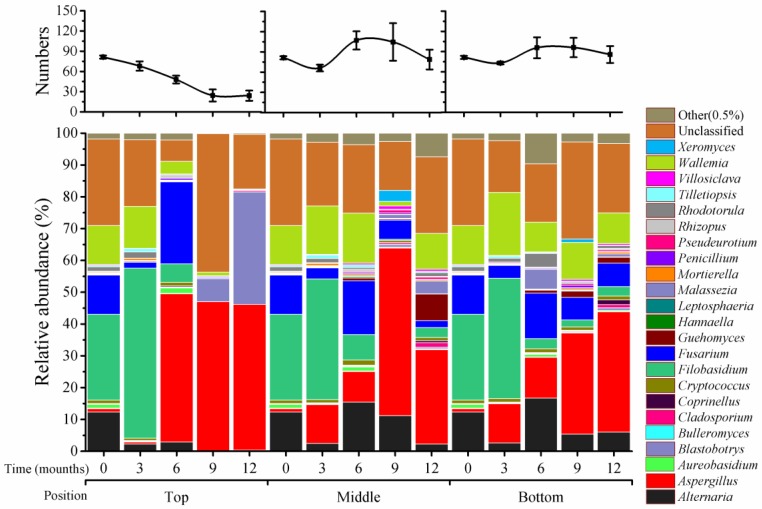
Distribution of fungi at the genus level in wheat stored for 0–12 months at different silo positions. Top, middle, and bottom indicate silo positions. The numbers 0, 3, 6, 9, and 12 represent the storage times in months in silos. The top panel represents the variation in the number of classified genera, the values represent the means of three replicates with the standard deviation (SD); the bottom panel represents the distribution of fungi at the genus level.

There were 81 genera that could be classified, and 21 genera that were unclassified at T_0_ in the fungal community. Substantial variations were detected in different positions of silos and at different storage times (Figure 2). At top positions, the genus numbers were gradually reduced from 81 (time 0), to 68 (3 months), 48 (6 months), and 24 (9 months), and maintained at 24 by 12 months; this was a 3.4-fold reduction between 0 and 9 months. However, in the middle and bottom positions, genus numbers were substantially increased at 6 (middle: 107; bottom: 96) and 9 months (middle: 105; bottom: 96), after a reduction at 3 months in middle (66) and bottom (73) positions. The numbers were maintained at a high level at 12 months (middle; 79; bottom, 86). 

There were four predominant genera at T_0_: *Filobasidium* (27%), *Fusarium* (12%), *Alternaria* (12%), and *Wallemia* (12%). These four genera displayed different patterns during storage. *Filobasidium* increased at 3 months (38% to 54%) and then declined sharply from 6 to 12 months (0% to 3%) in all three positions. *Fusarium* was reduced at 3 months (2% to 4%) and increased at 6 months (17% to 26%) in all positions; at 9 and 12 months, this genus accounted for only about 0.3% in top positions, 2% to 6% in the middle, and 7% in bottom positions. *Alternaria* and *Wallemia* had very low abundance (about 1%) at 9 to 12 months in top positions and retained quite high levels (2% to 17%) in the middle and bottom positions.

Four secondary genera, *Aspergillus*, *Aureobasidium*, *Cryptococcus*, and *Rhodotorula*, each accounted for about 1% at T_0_. Among them, *Aspergillus* had substantial increases of its relative abundance in all positions during storage; it remained at 1% at 3 months and increased at 6, 9, and 12 months (by about 47%) in top positions. In the middle and bottom positions, this genus had steady increases at 3 (10%), 6 (12%), 9 (30%), and 12 (53%) months. The remaining three genera remained constant during the entire storage period.

*Blastobotrys* varied significantly only in the top positions. The relative abundance of this genus was about 0.01% at T_0_, and at 3 and 6 months, but increased at 9 months (7%) and 12 months (35%) (Figure 2).

Unclassified fungi accounted for 27% of all fungi and the remaining 73 genera that were classified together accounted for only about 6% at T_0_. Unclassified fungi varied in top positions and were at a high proportion in middle and bottom positions. Overall, the 73 fungi had low relative abundance during the storage period.

### 2.4. Fungal Variation in Species across Storage Time at Different Silo Positions

**Figure 3:**
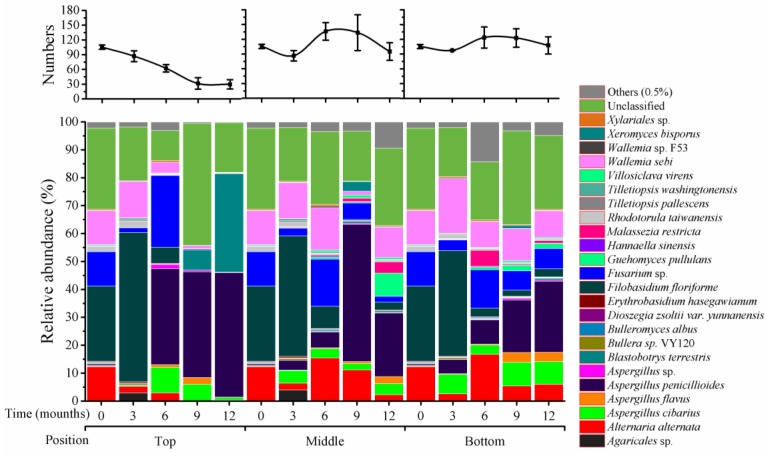
Distribution of fungi at the species level in wheat stored for 0–12 months at different silo positions. Top, middle, and bottom indicate silo positions. The numbers 0, 3, 6, 9, and 12 represent the storage times in months in silos. The top panel represents the variation of the number of classified species, the values represent the means of three replicates with the standard deviation (SD); the bottom panel represents the distribution of fungi at the species level.

As shown in Figure 3, there were 105 species that were classified at T_0_, and 41 species that were unclassified. In top positions, species numbers were gradually reduced at 3 (87), 6 (62), 9 (31), and 12 (30) months of storage. In middle positions, species increased at 6 (136) and 9 months (133) of storage and remained high at 12 months (95); similar variation was seen in bottom positions.

**Figure 4:**
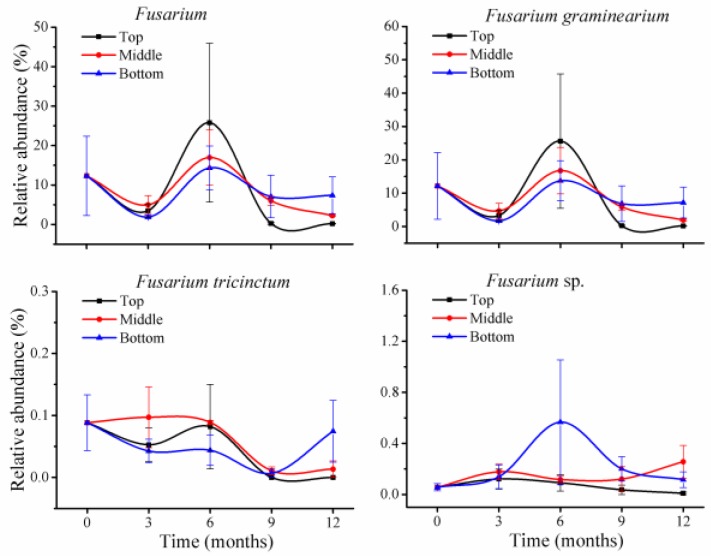
*Fusarium* sp. variations in stored wheat over storage time. The numbers 0, 3, 6, 9, and 12 represent the storage times in months in silos. The values represent the means of three replicates with the standard deviation (SD).

Among the 105 species, four predominant fungal species had greater than 10% of relative abundance at T_0_, including *Alternaria alternata* (12%), *Filobasidium floriforme* (27%), *Fusarium graminearum* (12%), and *Wallemia sebi* (12%). These relative abundances are almost the same as that of four genera presented above (Figure 2). This is because some genera either had only one species (*Alternaria* and *Filobasidium*) or other species within the genus had very low relative abundance; for instance, the genus *Fusarium* included three species, with *F. tricinctum* and another unclassified *Fusarium* sp. at less than 0.1% (Figure 4); in *Wallemia*, *Wallemia* sp. F53 was at 0.1% of relative abundance. Thus, variation patterns for these four species during storage are also the same as their genera shown above in Figure 2.

*Rhodotorula taiwanensis* had 1% and *Blastobotrys terrestris* had 0.1% of relative abundance at T_0_; these were the only species from these genera. Thus, variations of these two species during storage are the same as for their genera (Figure 2).

**Figure 5:**
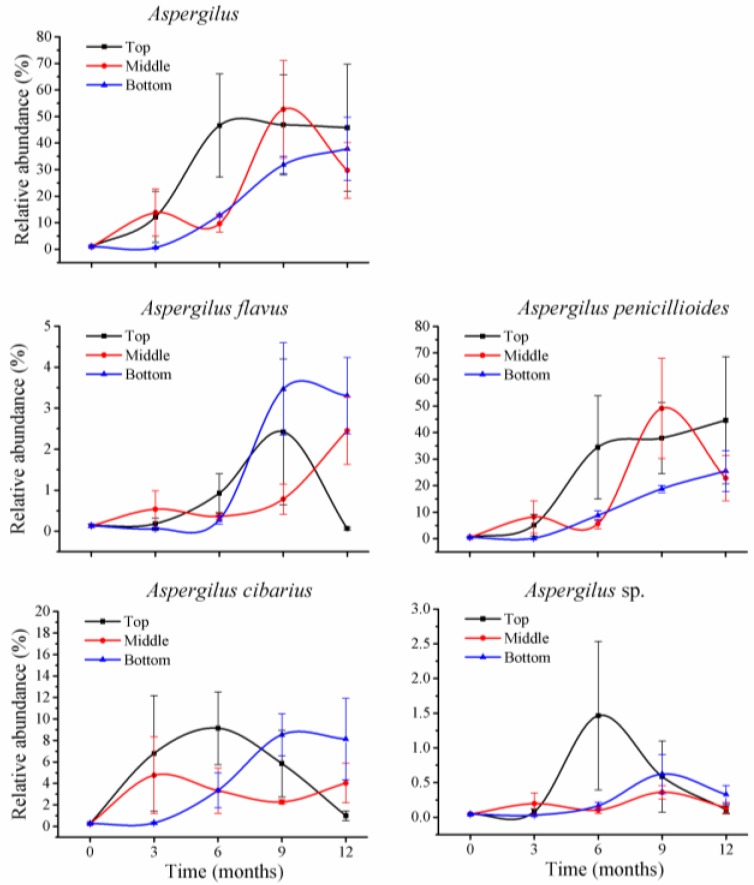
*Aspergillus* sp. variations in stored wheat over storage time. The numbers 0, 3, 6, 9, and 12 represent the storage times in months in silos. The values represent the means of three replicates with the standard deviation (SD).

Within *Aspergillus*, there were four species at less than 1% of relative abundance at T_0_ (Figure 5): *A. flavus* (0.1%), *A. cibarius* (0.3%), *A. penicillioides* (0.5%), and one unclassified species (0.05%). During storage, abundance of the first three species was variable; the relative abundance for *A. flavus* was 2% at 9 months in top positions, and at 12 months in middle positions, and 3% at 9 and 12 months in bottom positions, but was less than 1% of relative abundance in the remaining storage times. *A. cibarius* was at 9% at 6 months and 5% at 9 months in top positions, and 2 to 5% (middle positions) and 6 to 9% (bottom positions) during months 3 to 12.

### 2.5. Bacterial Variation across Storage Time at Different Silo Positions

Based on sequencing the bacterial V3V4 regions, nine phyla were detected and six of them were classified, including Proteobacteria, Firmicutes, Cyanobacteria, Chloroflexi, Bacteroidetes, and Actinobacteria (Figure S2). There were two predominant phyla, Firmicutes (6%) and Proteobacteria (92%), at T_0_, that had different patterns of variation during storage. In top and middle positions, Firmicutes had substantial increases at 9 months (top, 67%; middle 26%) and 12 months (top, 46%; middle, 53%), but at the same positions, Proteobacteria had reduced relative abundance at 9 (top, 31%; middle, 71%) and 12 months (top, 51%; middle, 47%). Cyanobacteria and Chloroflexi had disappeared from top positions by 12 months. 

There were 54 bacterial genera in the bacterial community that were classified, but around 90% of bacteria were unclassified (Figure S3). Among classified bacteria, there were three predominant genera, *Bacillus* (2%), *Paenibacillus* (4%), and *Pseudomonas* (3%) at T_0_. During storage, a substantial increase in relative abundance was seen at 9 and 12 months for *Bacillus* (36% and 4%), *Lactococcus* (10% and 7%), and *Staphylococcus* (14% and 28%) in top positions. In middle positions, *Lactococcus* accounted for 18% at 9 months and *Bacillus* for 48% at 12 months, whereas *Stenotrophomonas* accounted for 21% in 6 months at bottom positions.

From 60 classified bacterial species, three species, *Bacillus cereus*, *B. flexus*, and *Pseudomonas viridiflava*, each accounted for about 1% (Figure S4). More than 95% were unclassified. During storage in top and middle positions, *B. cereus* increased in relative abundance at 9 months (top, 36%; middle, 3%) and 12 months (top, 4%; middle, 15%); similarly, *Lactococcus garviease* also increased at these two storage times. *Bacillus flexus*, belonging to the phylum *Firmicutes*, had a slight increase at 9 months (4%) and significantly increased at 12 months (32%) in middle positions. Thus, only a few bacterial species varied substantially at certain silo positions during storage.

### 2.6. Correlation of Toxigenic Fusarium and Aspergillus Species with Other Fungi

Significant correlations were seen between toxigenic fungi *Fusarium* and *Aspergillus* species with other fungi during storage (Tables S3 and S4, Figures S5 and S6). For instance, variation of *F. graminearium* and *F. tricinctum* were significantly positively correlated with *A. alternata*, *Xylariales* sp., *Bulleromyces albus*, *Hannaella sinensis*, *Dioszegia zsoltii* var. *yunnanensis*, and *Pseudomonas viridiflava* in all silo positions at all storage times. Variation in *A. flavus* was significantly positively correlated with *Guehomyces pullulans*, *Villosiclava virens*, and *A. cibarius*, but negatively correlated with *Rhodotorula taiwanensis*. In addition, the variation of *A. cibarius* and *A. penicillioides* were significantly negatively correlated with *R. taiwanensis*.

### 2.7. DON and NIV Variation across Storage Time and Silo Positions

**Figure 6:**
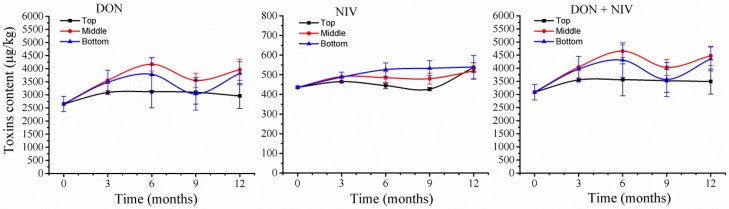
Variations in deoxynivalenol (DON) and nivalenol (NIV) toxins in stored wheat over storage time. Mycotoxin contents are the relative abundance in 5 g of stored wheat samples. The numbers 0, 3, 6, 9, and 12 represent the storage times in months in silos. The values represent the means of three replicates with the standard deviation (SD); each sample was measured twice.

Trichothecene mycotoxins were extracted from all 39 wheat samples and five mycotoxins, NIV, DON, 3A-DON, 15A-DON, and FX, were chemically analyzed. Two mycotoxins, DON (2652 μg/kg) and NIV (436 μg/kg), were detected in wheat grains at T_0_ (Figure 6). After three months of storage, the two mycotoxins accumulated to higher amounts in all three silo positions, with increases of 17–34% for DON (3090 to 3552 μg/kg) and 7–13% for NIV (465 to 490.5 μg/kg). Mycotoxin amounts varied at different silo positions during subsequent storage time points. In top positions, DON remained constant from 6 to 12 months (2957 μg/kg), whereas NIV declined at 9 months (427 μg/kg) and reached a peak at 12 months (537 μg/kg). In the other two positions, DON increased at 6 months (middle, 4168 μg/kg; bottom, 3784 μg/kg), declined at 9 months (middle, 3554 μg/kg; bottom, 3039 μg/kg), and increased again at 12 months (middle, 3958 μg/kg; bottom, 3820 μg/kg); these variations indicated significant increases of 43% (bottom) to 57% (middle) at 6 months and 44% (bottom) to 50% (middle) at 12 months compared with T_0_. NIV had the highest amounts at 12 months in all positions, with increases of 19–24%. Thus, the two mycotoxins increased during storage, but with different patterns, and DON increased more than NIV.

### 2.8. Aflatoxin Variations across Storage Time at Different Silo Positions

**Figure 7:**
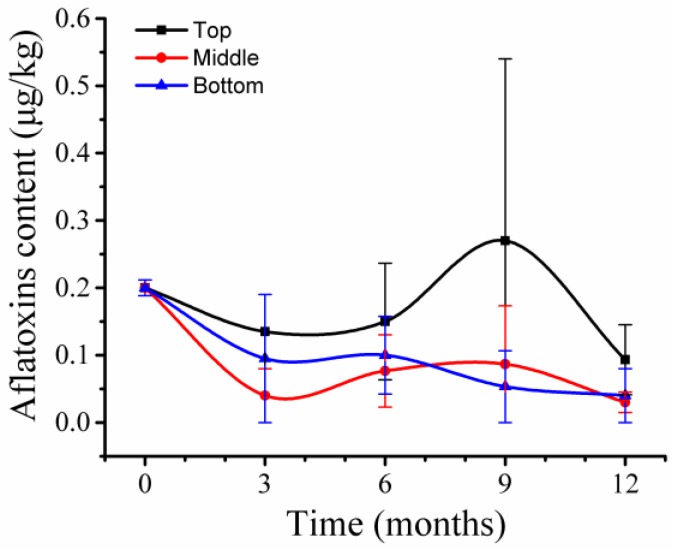
Aflatoxin variations in stored wheat over storage time. Mycotoxin contents are the relative abundance in 2 g of stored wheat samples. The numbers 0, 3, 6, 9, and 12 represent the storage times in months in silos. The values represent the means of three replicates with the standard deviation (SD).

Aflatoxins were extracted from 39 wheat samples and four types of aflatoxins—AFB1, AFB2, AFG1, and AFG2—were analyzed by LC/MS. Only AFG2 was detected at a level of 0.2 μg/kg in the samples at T_0_ (Figure 7). During storage, wheat samples at 9 months in top positions had the highest level of AFG2, 0.27 μg/kg, while other wheat samples had AFG2 at levels ranging from 0.03 μg/kg to 0.1 μg/kg. Thus, a low level of aflatoxins which are lower than regulatory limits [22] was present in wheat grains before and after storage. 

## 3. Discussion

Cereal grains, such as wheat, are currently widely stored under natural conditions with no controlled facilities in many developing countries, including China, where around 40% of grains are stored in small-scale silos in farmers’ houses. Investigation of microbiomes and associated mycotoxins of wheat grains in small-scale silos at room temperature may provide information that can be used to develop measures to prevent mycotoxins from entering food/feed chains. Metagenomic analyses of fungal ITS2 sequences and bacterial V3V4 sequences from microbiomes revealed the presence of at least 105 fungal species and 60 bacterial species on wheat grains before storage, which underwent substantial variations across times and silo positions during storage. This is the first known report of variation in microbiomes and associated mycotoxins on wheat grains during storage based on BIPES. 

Impacts of silo position on microbiome variation during storage were significant, especially for fungal communities. The numbers of fungi declined in top positions, but increased in the other two positions (Figure 1, Figure 2 and Figure 3); these changes then affected the relative abundances of individual species. It is likely that ventilation at top positions (around 30 cm from the top of the silo) is better than at middle and bottom positions; a silo microenvironment that includes ventilation may, thus, be unfavorable for growth and propagation of fungal species [6].

The toxigenic fungus *F. graminearium* was among the most variable species, with relative abundance that was reduced during the storage period; however, mycotoxins produced by *F. graminearium* increased. This phenomenon was particularly obvious at 3 months of storage in all three positions, with four- to six-fold reduction of fungi and 7–34% increase of mycotoxins. This variation may reflect complexity of the interaction between wheat and *F. graminearium*. Wheat cells could not metabolize *Fusarium* mycotoxins, and necrotrophic *Fusarium* fungi that already colonized wheat grains in the field continuously produced mycotoxins, leading to the accumulation of mycotoxins in wheat grains. Although the storage environment in top positions does not favor *Fusarium* growth, the reduction of colonizing *Fusarium* fungi on wheat grains took place gradually while mycotoxins steadily accumulated, giving rise to a steady increase of mycotoxins. After storage for 9 and 12 months in top positions, *Fusarium* fungi left on wheat grains were at very low levels, while amounts of mycotoxins remained constant. On the other hand, in middle and bottom positions, there was a high relative abundance of toxigenic fungi, and mycotoxins accordingly increased after 6 months, especially in the middle positions, where even higher amounts of mycotoxins were accumulated. These results provided more support for ventilation as an important factor for *Fusarium* fungi, whose relative abundance is closely associated with mycotoxin amounts in wheat grains during storage. Taken together, the present results suggested that several factors should be considered when storing wheat grains under natural conditions in regions similar to Wuhan: (i) heights of wheat grains in silos during storage should be about 70 cm or lower to provide a favorable ventilation environment; (ii) before storage, wheat grains should have levels of mycotoxins that are less than 750 μg/kg in order to avoid accumulation to the 1000 μg/kg limit set by regulatory office because mycotoxins could increase up to 34% during three months of storage; and (iii) if the heights are higher than 70 cm and storage is longer than three months, the amounts of mycotoxins in wheat before storage should be lower than 572 μg/kg (Figure 6).

*Aspergillus* fungi accounted for a low portion before storage and increased their relative abundance during storage. However, toxigenic *A. flavus* had low relative abundance and the predominant increase occurred for *A. cibarius*, a non-toxigenic *Aspergillus* species (Figure 5). Thus, aflatoxins were at very low levels before and during storage. These results indicated that during storage, toxigenic *Aspergillus* species are not predominant fungi, and aflatoxins are not the main mycotoxins in wheat grains under these conditions.

Bacteria in wheat grains detected by V3V4 sequencing were mainly taxonomically identified at the phylum level, and the vast majority of bacteria detected were unclassified at genus and species levels. Thus, it is currently difficult to analyze individual bacterial species and their impacts on the microbiome/mycotoxins on wheat grains during storage. These results implied that conventional methods of plating bacterial cells [23,24,25] might only detect a very small proportion of the active bacteria that are present on wheat grains. Therefore, more efforts may be required to characterize bacterial communities on wheat grains during storage using BIPES with other genome data of bacteria.

Correlation analyses suggest that some species of fungi and bacteria that can grow on wheat grains may have similar features, or that they interact each other to grow. How this interaction takes place requires further investigation.

It should be noted that the current study used a rinsing method [26,27] to isolate DNA for analysis of grain surface microbiome, and this may not isolate all fungal or bacterial species from the grains, especially for species from endophytes or cryptic infection. Thus, a higher fungal and bacterial diversity would be present in the stored wheat grains.

The current study revealed that, in wheat grains, *F. graminearum* was the predominant toxigenic fungal species, and the associated mycotoxins DON and NIV were the predominant mycotoxins. However, the fungal species substantially decreased, and mycotoxins significantly increased during storage. In the top positions of silos, these two mycotoxins and the fungi that produce them were all lower than at the other two silo positions. Aflatoxins and their producers, *Aspergillus* species, were low before and during storage. These results provide information for storage of wheat grains to reduce mycotoxin loads for food/feed products and for further studies on the mechanisms regulating variation of the microbiome and mycotoxins during storage of wheat.

## 4. Materials and Methods

### 4.1. Wheat Grain Samples

Wheat grains (variety: Wanmai68) were harvested in June 2015 from Anhui province in China, an area with frequent historical FHB epidemics. Grains were treated with ozone to kill insects as described previously [26,27], placed in three silos (I, II, and III) at 1000 kg per silo, and stored for a period of 12 months at room temperature in Wuhan. Each circular silo was 150 cm high (wheat grains reached 140 cm in each silo), with a diameter of 110 cm. Wheat samples obtained from the first day of storage were designated as T_0_ (1 August 2015), and one sample was obtained from each silo with a sampling apparatus according to a protocol [28]. After storage for 3, 6, 9 and 12 months, wheat samples were taken from three positions of each silo, top (110 cm from bottom, T), middle (70 cm from bottom, M), and bottom (30 cm from bottom, B), and the samples were named as 3T/M/B, 6T/M/B, 9T/M/B, and 12T/M/B. In each silo position, five samples were taken and combined as one sample (1 kg), giving rise to one sample for each silo position at one time. In total, 39 wheat samples were obtained and stored at −20 °C until use.

### 4.2. DNA Extraction from Microbiomes of Wheat Grains

One hundred grams of wheat grains from each sample were washed in 100 mL sterilized water. The resulting liquid was collected and vacuum-filtered through 0.22 um filters [27]. The filters containing microbiomes were placed in 5 mL tubes and stored at −20 °C. DNA from the microbiome on the filters was extracted with the MoBio Power Water DNA Isolation Kit (MoBio Laboratories, Inc., Carlsbad, CA, USA) according to the manufacturer’s instructions. The extracted DNA was suspended in sterile deionized water, DNA quantity was measured by spectrophotometric quantification in a NanoDrop 1000 (Thermo Fisher Scientific, Inc., Newark, DE, USA), and DNA quality was assayed by agarose gel electrophoresis. Extracted DNA was stored at −80 °C.

### 4.3. PCR Amplification and Sequencing of Fungal ITS2 and Bacterial V3V4

The universal fungal primers (annealing temperature of 50 °C) ITS3 (5′-GCATCGATGAAGAACGCAGC-3′) and ITS4 (5′-TCCTCCGCTTATTGATATGC-3′) were used to amplify ITS2 regions of fungal rDNA. Bacterial universal primers (annealing temperature of 50 °C) 341F (5′-ACTCCTACGGGAGGCAGCAG-3′) and 806R (degenerate primer) (5′-GGACTACHVGGGTWTCTAAT-3′) were used to amplify the V3V4 regions of bacterial rDNA. PCR reactions were carried out in a total reaction volume of 50 μL consisting of 5 μL KOD plus buffer, 5 μL dNTP, 2 μL MgSO_4_, 0.5 μM of forward and reverse primers, and 10 ng of template DNA. The PCR amplification program consisted of initial heating to 95 °C for 5 min, 35 cycles of denaturation at 95 °C for 20 s, annealing at 50 °C for 30 s, and extension at 68 °C for 60 s, followed by a 10 min extension at 68 °C. The AxyPrepTM DNA Gel Extraction Kit (Axygen, Jiangsu, Suzou, China) was used to purify the amplified products. Amplicons were quantified, and sequencing libraries were generated using NEB Next Ultra DNA library Prep Kit for Illumina (New England Biolabs, Inc., Ipswich, MA, USA). The libraries were subsequently sequenced on an Illumina MiSeq PE250 platform at the Beijing Genomics Institute (BGI, Wuhan, China).

### 4.4. DNA Sequence Analysis and Bioinformatics

FLASH was used to merge the paired-end reads to a tag from the high quality clean reads. The tags were clustered to operational taxonomic unit (OTU) by scripts in the program USEARCH (v7.0.1090, Sonoma, CA, USA, 2014), detailed as follows: (i) tags were clustered into OTUs with a 97% threshold using UPARSE, and unique, representative OTU sequences were obtained; (ii) chimeras were filtered out using UCHIME (v4.2.40, Sonoma, CA, USA, 2017), and the 16S rDNA and ITS2 sequences were screened for chimeras by separate mapping to the gold database (v20110519) or UNITE (v20140703), and de novo chimera detection was done for 18S rDNA sequences; and (iii) all tags were mapped to each OTU representative sequence using USEARCH GLOBAL, and then the tag numbers of each OTU in each sample were summarized in an OTU abundance table. OTU representative sequences were taxonomically classified using Ribosomal Database Project Classifier (RDP, v.2.2, East Lansing, MI, USA, 2016) trained on the Greengenes database, using 0.8 confidence values as cutoffs. 16S rDNA was used for bacterial and archaeal communities (Greengenes, V201305, Berkeley, CA, USA, 2013), and ITS was used for the fungal community (UNITE, Version6, Vanemuise, Tartu, Estonia). Alpha diversity was used to analyze complexity of species diversity for a sample based on several indices, including observed species, Chao1, ACE, and Shannon and Simpson indexes. Beta diversity analysis was used to evaluate differences of samples in terms of their species complexity. Beta diversity analysis was conducted in QIIME (v1.80, Twin Cities, MN, USA, 2017). Correlation analysis was performed using Origin (v9.1) in the microbiome. Differences were considered significant at *p* < 0.05.

### 4.5. Quantification of Trichothecenes

Stored wheat grains (5 grams) were ground to a powder. Crude mycotoxins were extracted as previously described [29,30]. For each sample, ground grains were extracted three times with 20 mL extraction solvent (acetonitrile/water = 84:16, *v/v*). Extracts were cleaned-up using a silica gel cartridge and a MultiSep 211 column (Romer Labs, Washington, MO, USA) before analysis. Gas chromatography-mass spectrometry analyses (GC-MS) of DON, NIV and their derivatives were performed as described previously [28]. Standards for DON, 3-acetyldeoxynivalenol (3A-DON), 15-acetyldeoxynivalenol (15A-DON), fusarenon-X (FX), and NIV were purchased from Sigma (St. Louis, MO, USA).

### 4.6. Determination of Aflatoxins

Ground wheat samples (2 grams) were placed into 50 mL centrifuge tubes, and 8 mL of acetonitrile/water/methanoic acid solution (80:19:1, *v/v/v*) were added for extraction of crude mycotoxins as previously described [31,32,33]. After vortexing for 2 min, samples were subjected to ultrasonication for 40 min, followed by centrifuging at 4000 rpm for 5 min. Then, 2 mL of the supernatant were transferred into a screw cap test tube containing 150 mg anhydrous MgSO_4_ and 1 mL hexane. The mixture was immediately shaken for 2 min. The hexane layer was removed after centrifugation at 4000 rpm for 5 min, and the remaining solution was evaporated to dryness at 50 °C under a stream of nitrogen gas. The residue was re-dissolved in a 1 mL acetonitrile/water solution (20:80, *v/v*). The mixture was filtrated through a 0.22 μm nylon filter and four aflatoxins—aflatoxin B1 (AFB1), aflatoxin B2 (AFB2), aflatoxin G1 (AFG1), and aflatoxin G2 (AFG2)—were measured using ultra-high performance liquid chromatography-tandem mass spectrometry (UHPLC-MS/MS) [31,32,33]. Standards for AFB1, AFB2, AFG1, and AFG2 were purchased from Alexisa (San Diego, CA, USA).

### 4.7. Climatic Data

During the storage period, climatic data, such as temperature (°C) and RH (Relative humidity %), were recorded monthly in the regions of the experiment. Daily mean temperature is the average value of temperature at 2:00, 8:00, 14:00, and 20:00 each day. Monthly mean temperature is the average value of daily mean temperature in 1 month. Three monthly mean temperature is the average value of daily mean temperature in 3 months. The calculation of daily mean RH, monthly mean RH and three monthly mean RH is similar with temperature. The temperature and RH in storage areas were shown in Figure S7.

### 4.8. Statistical Analyses

The correlation coefficient analysis of fungi was conducted in OriginPro 9.1 (OriginLab Corporation, Northampton, MA, USA) software that was evaluated by the Pearson correlation. Differences were considered significant at *p* < 0.05.

## Supplementary Materials

The following are available online at http://www.mdpi.com/2072-6651/10/5/171/s1, Figure S1: PCR amplicons of ITS2 and V3V4 in wheat samples during storage, Figure S2: Distribution of bacteria at the phylum level in wheat stored for 0–12 months at different silos positions, Figure S3: Distribution of bacteria at the genus level in wheat stored for 0–12 months at different silos positions, Figure S4: Distribution of bacteria at the species level in wheat stored for 0–12 months at different silo positions, Figure S5: Correlation of *Fusarium* sp. abundance with other species, Figure S6: Correlations of *Aspergillus* sp. abundance with other species, Figure S7: Temperature and relative humidity in Wuhan Storage areas, Table S1: Statistics of ITS sequences, Table S2: Statistics of V3V4 sequences, Table S3: The relationships of *Fusarium* sp. with other species, Table S4: The relationships of *Aspergillus* sp. with other species.
