# Supplementary material for: Variation in the Microbiome, Trichothecenes, and Aflatoxins in Stored Wheat Grains in Wuhan, China
Source: Toxins (Basel). 2018 Apr 24;10(5):171. doi: 10.3390/toxins10050171 (PMC5983228; doi:10.3390/toxins10050171)
Supplement: Supplementary file 1 [file toxins-10-00171-s001.pdf]

Supplementary Materials: Variation in the microbiome, trichothecenes, and aflatoxins in stored wheat grains in Wuhan, China

Qing-Song Yuan, Peng Yang, Ai-Bo Wu, Dong-Yun Zuo, Wei-Jie He, Mao-Wei Guo, Tao Huang, He-Ping Li and Yu-Cai Liao

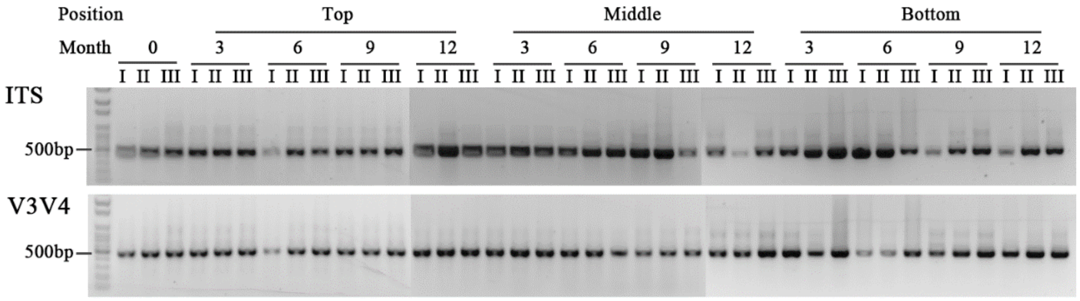

Figure S1. PCR amplicons of ITS2 and V3V4 in wheat samples during storage. I, II, and III represent three repeats (silos) of the experiments, 0, 3, 6, 9, and 12 represent the storage times in months in silos.

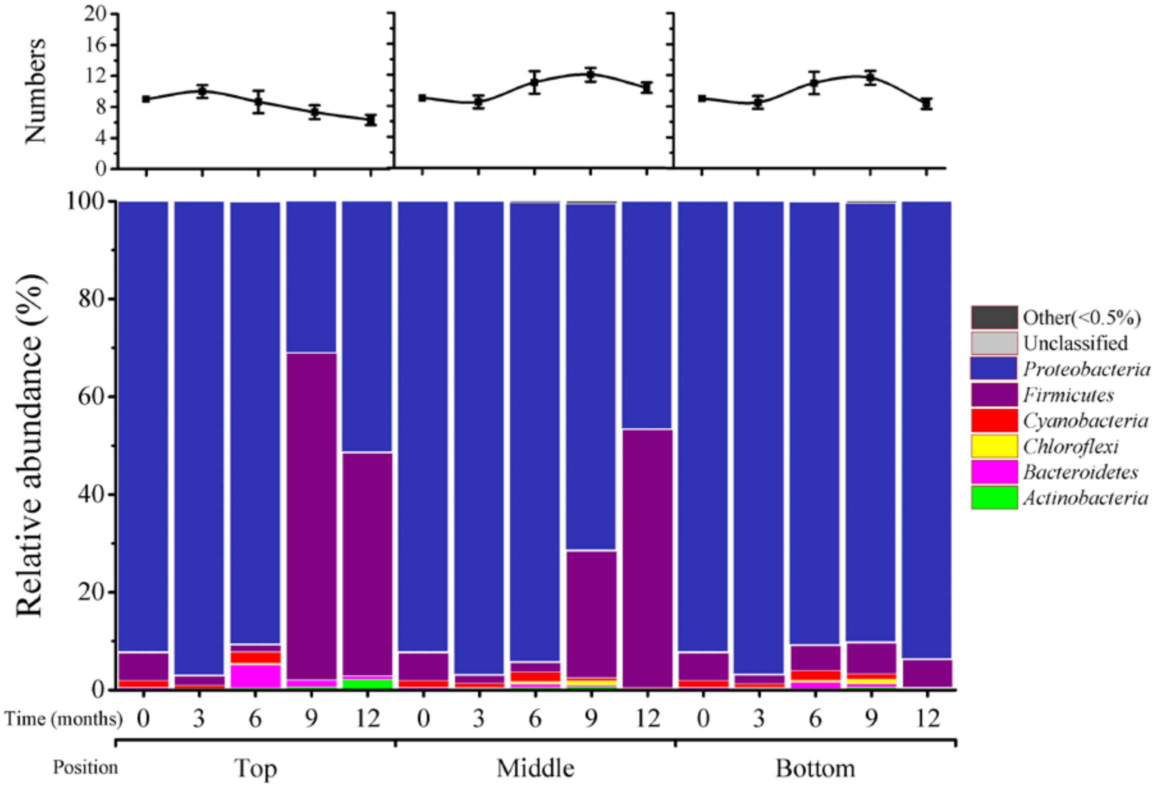

Figure S2. Distribution of bacteria at the phylum level in wheat stored for 0–12 months at different silos positions. Top, middle, and bottom indicate silo positions. 0, 3, 6, 9, and 12 represent the storage times in months in silos. The top panel represents the variation of the number of classified phyla, the values represent the means of three replicates with the standard deviation (SD); the bottom panel represents the distribution of bacteria at the phylum level.

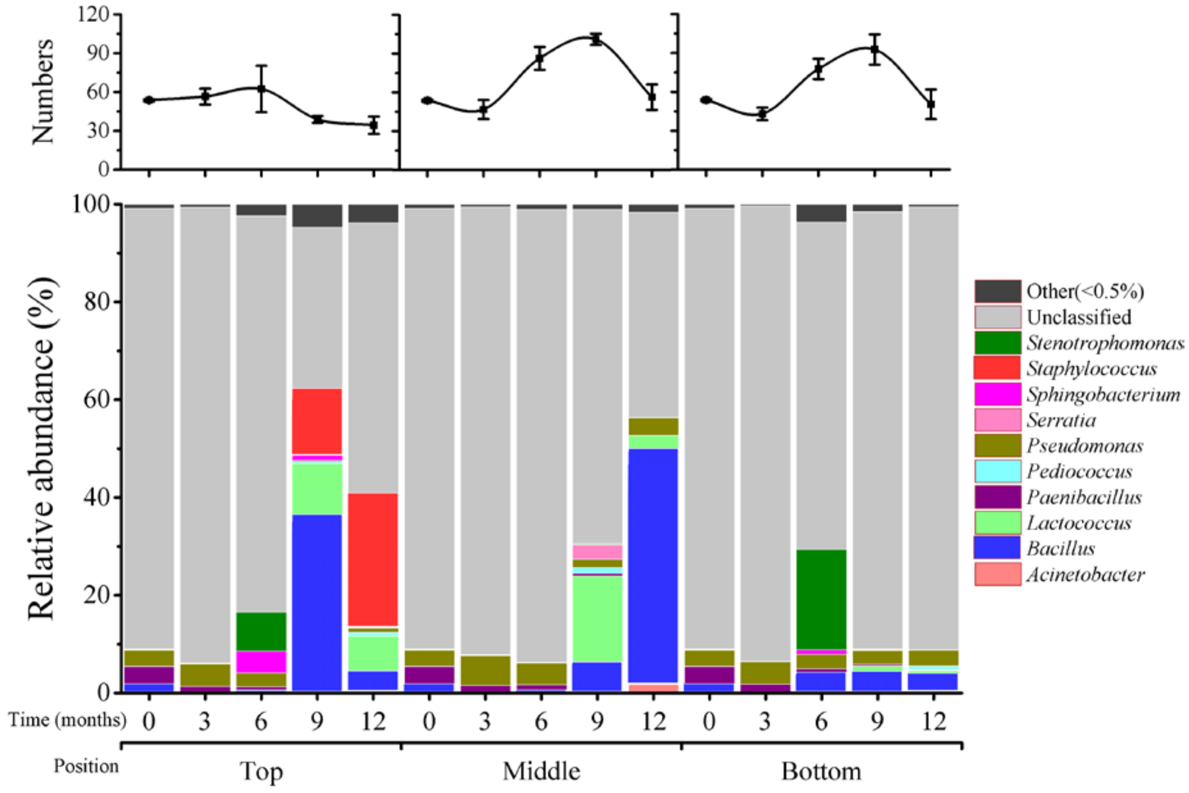

Figure S3. Distribution of bacteria at the genus level in wheat stored for 0–12 months at different silos positions. Top, middle, and bottom indicate silo positions. 0, 3, 6, 9, and 12 represent the storage times in months in silos. The top panel represents the variation of the number of classified genera, the values represent the means of three replicates with the standard deviation (SD); the bottom panel represents the distribution of bacteria at the genus level.

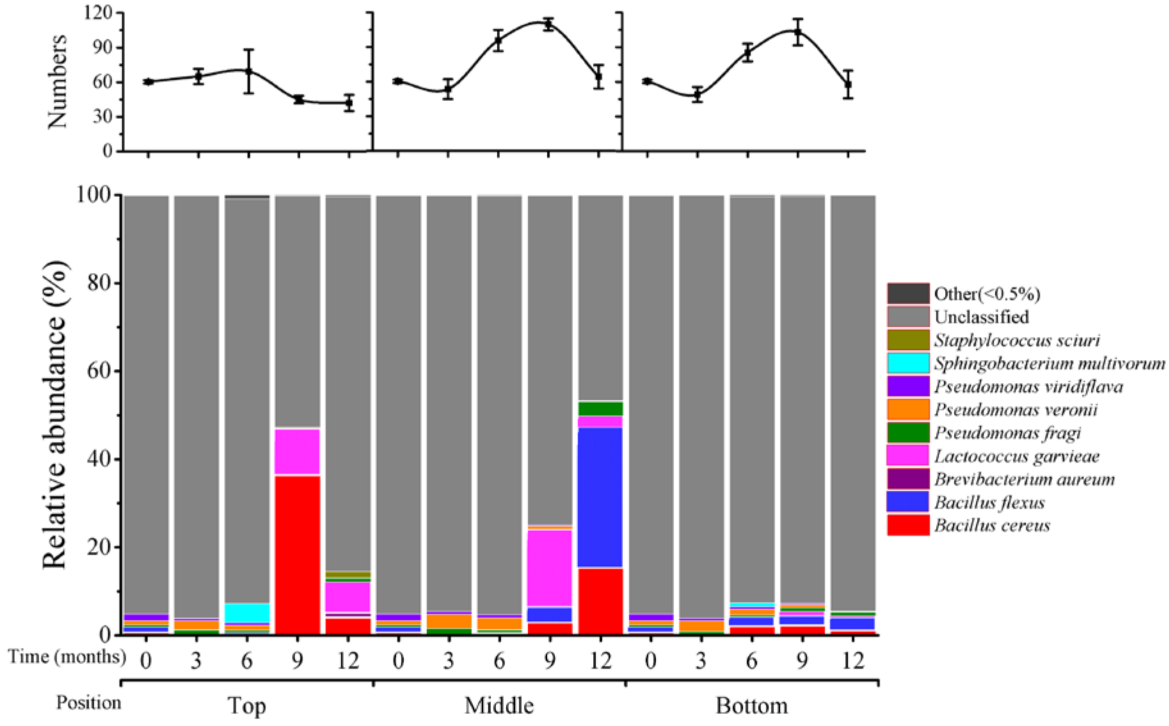

Figure S4. Distribution of bacteria at the species level in wheat stored for 0–12 months at different silo positions. Top, middle, and bottom indicate silo positions. 0, 3, 6, 9, and 12 represent the storage times in months in silos. The top panel represents the variation of the number of classified species, the values represent the means of three replicates with the standard deviation (SD); the bottom panel represents the distribution of bacteria at the species level.

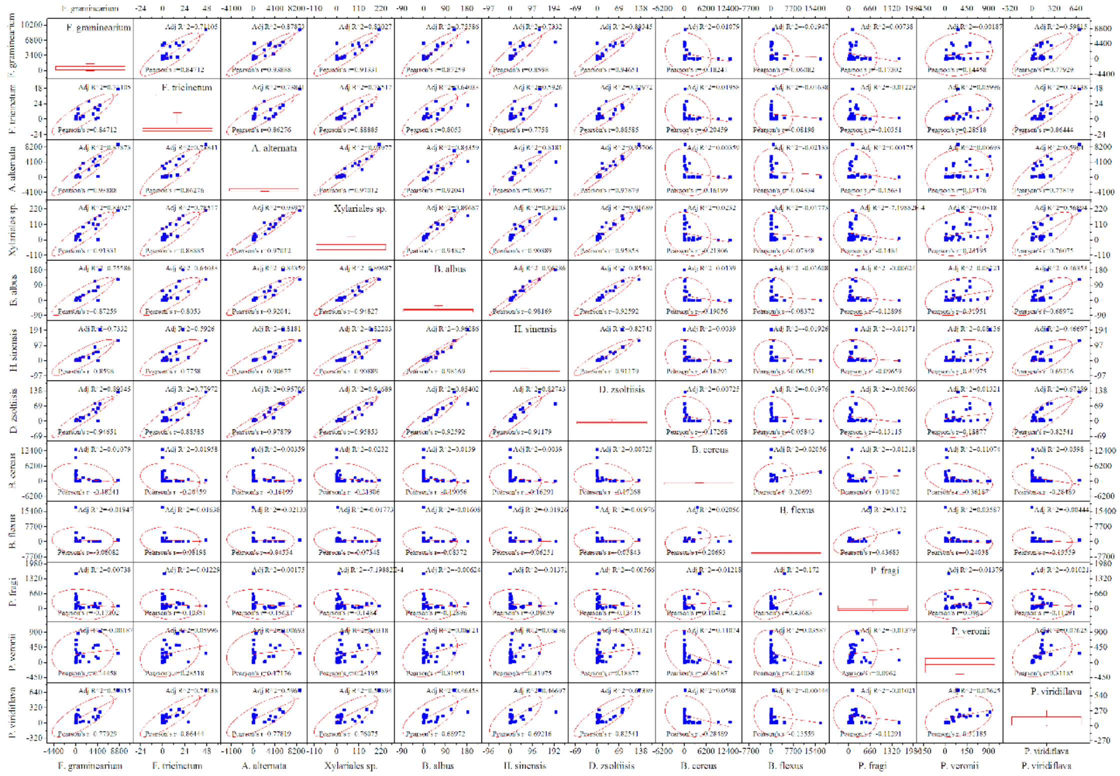

Figure S5. Correlation of *Fusarium* sp. abundance with other species. Static data of relative abundance of each fungus species at the top, middle, and bottom silo positions, and at different storage time points, and analysis of their relationships. The circular diagrams and flat circles represent lower and higher correlations, respectively; the sample spot distribution represents the significance.

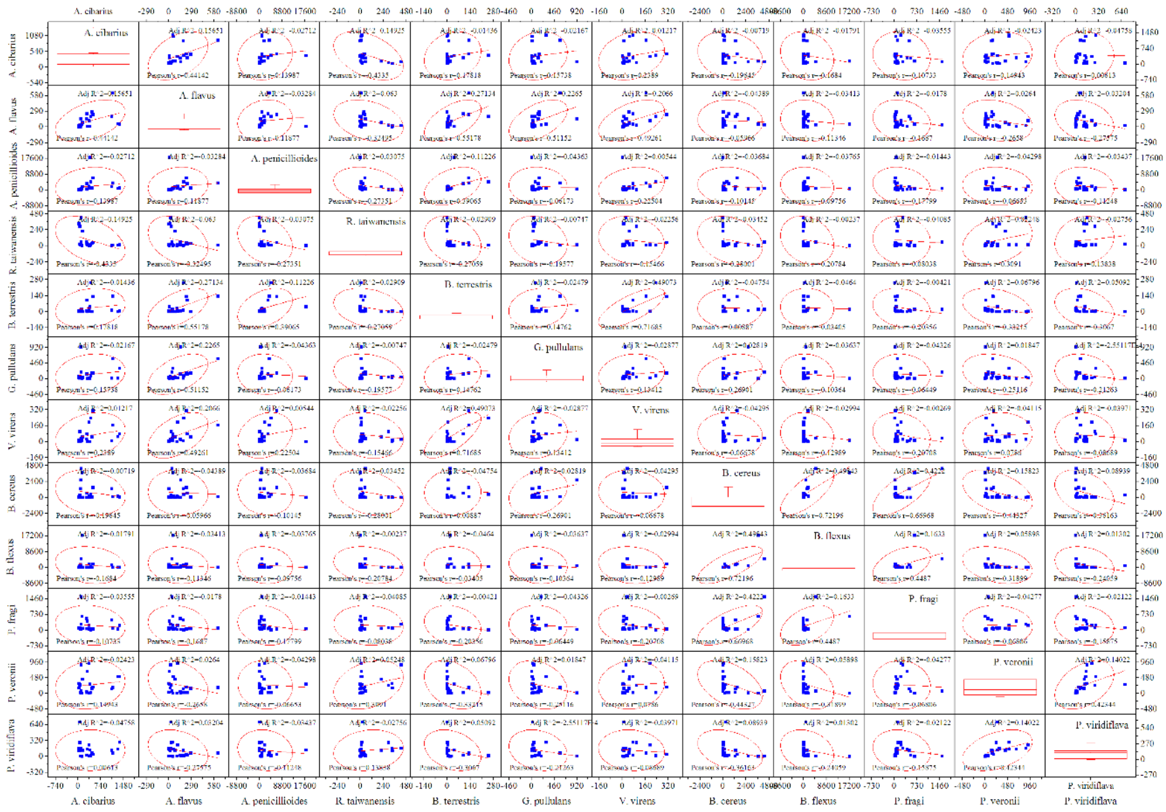

Figure S6. Correlations of *Aspergillus* sp. abundance with other species. Static data of relative abundance of each fungus species at the top, middle, and bottom silo positions, and at different storage time points, and analysis of their relationships. The circular diagrams and flat circles represent lower and higher correlations, respectively; the sample spot distribution represents the significance.

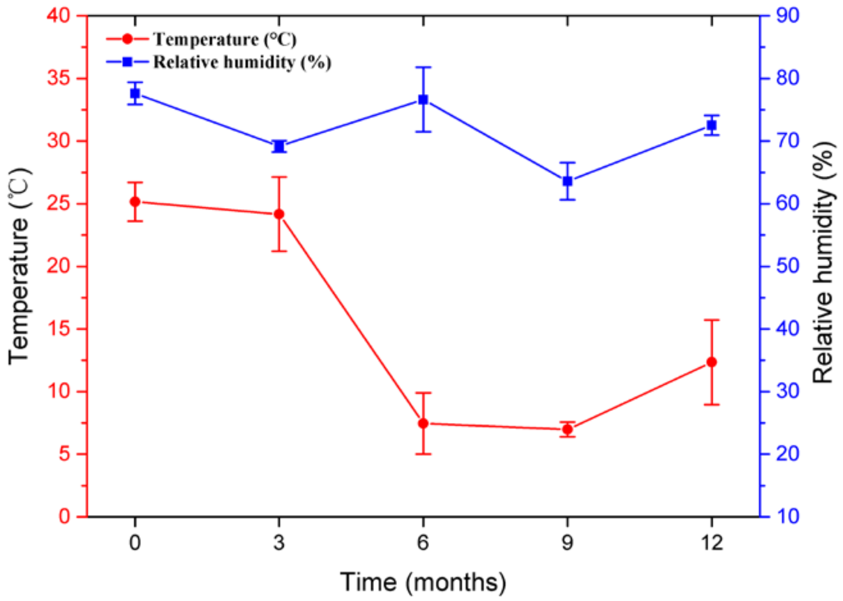

Figure S7. Temperature and relative humidity in Wuhan Storeage areas.

Table S1. Data Statistics of ITS sequence

Sample Name	Reads Length (bp)	Clean Reads	Read Utilization Ratio (%)	Tags Without Primer	Tag Utilization Ratio (%)	Average Length And SD	OTU Number
0I	293:294	24243*2	95.01	21863	90.18	366/24	200
0II	299:294	25397*2	90.77	22709	89.42	365/21	202
0III	298:294	30735*2	87.7	28470	92.63	308/21	186
3BI	297:294	30091*2	95.31	25573	85.02	372/21	221
3BII	296:294	30252*2	96.38	27330	90.31	366/12	151
3BIII	294:294	29859*2	91.4	26982	90.3	368/26	186
3MI	297:294	30091*2	95.31	25583	85.02	371/21	241
3MII	296:294	30252*2	96.38	27320	90.31	367/12	197
3MIII	294:294	29859*2	91.4	26962	90.3	368/26	143
3TI	297:294	30091*2	95.31	25583	85.02	371/21	152
3TII	296:294	30252*2	96.38	27220	90.31	367/12	121
3TIII	294:294	29859*2	91.4	26162	90.3	368/26	183
6BI	300:294	30784*2	81.13	28199	91.6	336/33	266
6BII	298:294	30960*2	84.4	27930	90.21	328/31	289
6BIII	300:299	23851*2	96.72	21087	88.41	370/15	196
6MI	300:294	30114*2	95.84	26903	89.34	367/13	283
6MII	299:294	30565*2	88.75	27469	89.87	330/34	279
6MIII	297:294	31142*2	84.96	27660	88.82	323/31	370
6TI	293:294	29816*2	97.29	26599	89.21	367/12	104
6TII	296:299	31842*2	77.95	28864	90.65	347/28	129
6TIII	297:293	30297*2	94.75	27166	89.67	366/12	71
9BI	294:294	31271*2	78.48	28252	90.35	327/29	334
9BII	299:300	29359*2	92.2	26744	91.09	369/23	195
9BIII	296:300	29580*2	95.8	26099	88.23	364/20	404
9MI	296:294	30588*2	81.69	27410	89.61	335/32	437
9MII	295:298	32024*2	60.79	29151	91.03	333/24	98
9MIII	293:294	31424*2	71.4	28064	89.31	333/31	466
9TI	294:293	23641*2	91.25	21382	90.44	359/28	69
9TII	293:300	29975*2	97.94	27295	91.06	370/8	20
9TIII	298:300	29380*2	95.2	27062	92.11	365/14	48
12BI	300:294	30244*2	44.32	27138	89.73	340/31	235
12BII	298:299	25496*2	96.39	22604	88.66	367/15	184
12BIII	294:299	29935*2	96.81	26925	89.94	365/17	349
12MI	293:300	27571*2	95.1	24367	88.38	367/20	392
12MII	299:299	29435*2	96.93	26695	90.69	369/13	196
12MIII	296:299	26063*2	95.89	20882	80.12	367/18	249
12TI	294:300	30779*2	91.34	28080	91.23	330/15	55
12TII	293:299	30119*2	98.15	27360	90.84	368/8	31
12TIII	297:299	29697*2	83.88	26604	89.58	338/22	57
Average	293:300	29409*2	89.44	26324	89.47	355/21	205

T, M, and B represent the top, middle, and bottom silo positions, respectively; I, II, and III represent the repeat silos of the experiments. Read lengths are the range in each sample, the number of pairs of clean reads following removal of low quality reads. Tags are assembled from clean read pairs, and the tags were clustered to Operational Taxonomic Unit (OTU) by scripts in the program USEARCH (v7.0.1090) at a 97% threshold value.

Table S2. Data Statistics of V3V4 sequence

Sample Name	Reads Length (bp)	Clean Reads	Read Utilization Ratio (%)	Tags Without Primer	Tag Utilization Ratio (%)	Average Length And SD	OTU Number
0I	297:300	30205*2	92.57	29874	98.9	428/3	89
0II	296:300	30601*2	92.17	30271	98.92	428/2	85
0III	295:300	30629*2	92.29	30303	98.94	427/5	94
3BI	294:300	30634*2	92.89	30477	98.83	427/3	107
3BII	293:300	31044*2	91.27	30641	98.7	428/3	57
3BIII	300:300	30165*2	91.59	29654	98.97	426/2	66
3MI	294:300	30634*2	92.89	30277	98.83	428/3	104
3MII	293:300	31044*2	91.27	30641	98.7	428/3	122
3MIII	300:300	30165*2	91.59	29854	98.97	428/2	76
3TI	294:300	30634*2	92.89	30077	98.83	428/3	77
3TII	293:300	31044*2	91.27	30841	98.7	428/3	142
3TIII	300:300	30165*2	91.59	29854	98.97	428/2	109
6BI	297:299	30096*2	93.04	29758	98.88	428/4	118
6BII	298:300	30163*2	91.51	29795	98.78	427/5	207
6BIII	299:300	30050*2	92.08	29730	98.94	428/4	66
6MI	295:299	30864*2	92.74	30494	98.8	427/5	162
6MII	296:299	30552*2	93.12	30183	98.79	427/5	154
6MIII	299:297	30733*2	93.5	30388	98.88	426/6	195
6TI	298:297	30581*2	93.11	30267	98.97	426/7	167
6TII	293:299	30495*2	92.42	30034	98.49	427/4	163
6TIII	294:299	30564*2	93.94	30201	98.81	428/3	86
9BI	298:299	30806*2	92.33	30475	98.93	428/4	153
9BII	299:299	30080*2	93.38	29717	98.79	428/4	180
9BIII	300:299	29904*2	93.39	29504	98.66	428/3	62
9MI	296:298	30864*2	92.2	30399	98.49	428/4	170
9MII	297:296	30925*2	91.53	30571	98.86	427/6	238
9MIII	297:298	30645*2	92.5	30304	98.89	428/1	49
9TI	293:298	30474*2	91.42	29989	98.41	426/6	239
9TII	294:298	30355*2	93.01	29936	98.62	428/4	200
9TIII	295:298	30509*2	92.7	30155	98.84	429/1	60
12BI	298:298	30338*2	91.67	29991	98.86	428/2	87
12BII	299:298	30701*2	91.74	30298	98.69	428/2	130
12BIII	300:298	30694*2	92.36	30182	98.33	427/5	59
12MI	295:297	30577*2	92.36	30267	98.99	428/1	60
12MII	296:297	30372*2	93.33	29990	98.74	429/1	64
12MIII	297:297	30206*2	93.59	29890	98.95	428/2	72
12TI	300:297	30570*2	92.34	30262	98.99	428/3	141
12TII	293:297	30843*2	92.02	30418	98.62	428/2	102
12TIII	294:297	30923*2	92.27	30620	99.02	428/1	38
Average	293:300	30535*2	92.41	30169	98.80	427/3	117

T, M, and B represent the top, middle, and bottom silo positions, respectively; I, II, and III represent the repeat silos of the experiments. Read lengths are the range in each sample, the number of pairs of clean reads following removal of low quality reads. Tags are assembled from clean read pairs, and the tags were clustered to Operational Taxonomic Unit (OTU) by scripts in the program USEARCH (v7.0.1090) at a 97% threshold value

Table S3. The relationships of *Fusarium* sp. with other species

		F. graminearium	*F. tricinctum*	*A. alternata*	*Xylariales* **sp.**	*B. albus*	*H. sinensis*	*D. zsoltiisis*	*B. cereus*	*B. flexus*	*P. fragi*	*P. veronii*	*P. viridiflava*
F. graminearium	Pearson r	1	0.84712	0.93888	0.91331	0.87259	0.8598	0.94651	-0.18241	-0.06082	-0.17302	0.14458	0.77929
	Sig.	--	2.21E-13	0	0	5.77E-15	3.93E-14	0	0.2304	0.69147	0.25571	0.34335	2.87E-10
F. tricinctum	Pearson r	0.84712	1	0.86276	0.88885	0.8053	0.7758	0.88585	-0.20459	-0.08198	-0.10351	0.28518	0.86444
	Sig.	2.21E-13	--	2.55E-14	4.44E-16	2.56E-11	3.87E-10	6.66E-16	0.17762	0.59241	0.49862	0.05759	2.00E-14
A. alternata	Pearson r	0.93888	0.86276	1	0.97012	0.92041	0.90677	0.97879	-0.16199	-0.04334	-0.15631	0.17176	0.77819
	Sig.	0	2.55E-14	--	0	0	0	0	0.28773	0.7774	0.30517	0.25923	3.15E-10
***Xylariales* sp.**	Pearson r	0.91331	0.88885	0.97012	1	0.94827	0.90889	0.95853	-0.21306	-0.07348	-0.1484	0.23195	0.76075
	Sig.	0	4.44E-16	0	--	0	0	0	0.15995	0.63144	0.3306	0.12523	1.33E-09
B. albus	Pearson r	0.87259	0.8053	0.92041	0.94827	1	0.98169	0.92592	-0.19056	-0.08372	-0.12896	0.31951	0.68972
	Sig.	5.77E-15	2.56E-11	0	0	--	0	0	0.20989	0.58454	0.39853	0.0324	1.60E-07
H. sinensis	Pearson r	0.8598	0.7758	0.90677	0.90889	0.98169	1	0.91179	-0.16291	-0.06251	-0.09659	0.31975	0.69216
	Sig.	3.93E-14	3.87E-10	0	0	0	--	0	0.28495	0.68332	0.52792	0.03226	1.39E-07
D. zsoltiisis	Pearson r	0.94651	0.88585	0.97879	0.95853	0.92592	0.91179	1	-0.17268	-0.05843	-0.13115	0.18877	0.82541
	Sig.	0	6.66E-16	0	0	0	0	--	0.25667	0.70303	0.3905	0.21427	3.05E-12
B. cereus	Pearson r	-0.18241	-0.20459	-0.16199	-0.21306	-0.19056	-0.16291	-0.17268	1	0.20693	0.10402	-0.36187	-0.28489
	Sig.	0.2304	0.17762	0.28773	0.15995	0.20989	0.28495	0.25667	--	0.1726	0.49652	0.01458	0.05785
B. flexus	Pearson r	-0.06082	-0.08198	-0.04334	-0.07348	-0.08372	-0.06251	-0.05843	0.20693	1	0.43683	-0.24038	-0.13559
	Sig.	0.69147	0.59241	0.7774	0.63144	0.58454	0.68332	0.70303	0.1726	--	0.0027	0.1117	0.37451
P. fragi	Pearson r	-0.17302	-0.10351	-0.15631	-0.1484	-0.12896	-0.09659	-0.13115	0.10402	0.43683	1	0.0962	-0.11291
	Sig.	0.25571	0.49862	0.30517	0.3306	0.39853	0.52792	0.3905	0.49652	0.0027	--	0.52961	0.46022
P. veronii	Pearson r	0.14458	0.28518	0.17176	0.23195	0.31951	0.31975	0.18877	-0.36187	-0.24038	0.0962	1	0.31185
	Sig.	0.34335	0.05759	0.25923	0.12523	0.0324	0.03226	0.21427	0.01458	0.1117	0.52961	--	0.03703
P. viridiflava	Pearson r	0.77929	0.86444	0.77819	0.76075	0.68972	0.69216	0.82541	-0.28489	-0.13559	-0.11291	0.31185	1
	Sig.	2.87E-10	2.00E-14	3.15E-10	1.33E-09	1.60E-07	1.39E-07	3.05E-12	0.05785	0.37451	0.46022	0.03703	--

Static data of relative abundance of each fungal species at the top, middle, and bottom silo positions and different storage time points; analysis of their relationships; Pearson's r represents the correlation coefficient, Sig. indicates the significance level, yellow background indicates a significant correlation at $p < 0.05$ level.

Table S4. The relationships of *Aspergillus* sp. with other species

		A. cibarius	*A. flavus*	*A. penicillioides*	*R. taiwanensis*	*B. terrestris*	*G. pullulans*	*V. virens*	*B. cereus*	*B. flexus*	*P. fragi*	*P. veronii*	*P. viridiflava*
A. cibarius	Pearson r	1	0.47478	0.12905	-0.39622	-0.07679	0.21689	0.27066	-0.00141	-0.05298	-0.04451	0.04837	-0.10346
	Sig.	--	9.83E-04	0.3982	0.00705	0.61609	0.15241	0.07213	0.99266	0.72959	0.77157	0.75237	0.49885
A. flavus	Pearson r	0.47478	1	0.13261	-0.27937	-0.05236	0.54666	0.52766	0.14974	-0.0025	-0.10076	-0.23991	-0.2362
	Sig.	9.83E-04	--	0.38519	0.0631	0.73268	1.02E-04	1.96E-04	0.3262	0.98699	0.51017	0.11243	0.11826
A. penicillioides	Pearson r	0.12905	0.13261	1	-0.29848	0.15725	-0.03953	0.1367	0.02583	-0.05143	-0.18416	-0.2124	-0.22509
	Sig.	0.3982	0.38519	--	0.04641	0.30224	0.79656	0.37056	0.86624	0.73725	0.22589	0.16128	0.13711
R. taiwanensis	Pearson r	-0.39622	-0.27937	-0.29848	1	-0.12669	-0.10177	-0.03587	-0.2681	-0.17781	-0.06562	0.37102	0.10461
	Sig.	0.00705	0.0631	0.04641	--	0.40693	0.5059	0.81503	0.07498	0.24259	0.66845	0.0121	0.49407
B. terrestris	Pearson r	-0.07679	-0.05236	0.15725	-0.12669	1	-0.08084	-0.09191	0.12367	-0.04473	-0.05954	-0.16432	-0.14525
	Sig.	0.61609	0.73268	0.30224	0.40693	--	0.59759	0.54819	0.41831	0.77048	0.69766	0.28075	0.34109
G. pullulans	Pearson r	0.21689	0.54666	-0.03953	-0.10177	-0.08084	1	0.24724	0.0087	-0.0163	-0.01069	-0.16006	-0.15465
	Sig.	0.15241	1.02E-04	0.79656	0.5059	0.59759	--	0.10154	0.95479	0.91535	0.94444	0.29357	0.31042
V. virens	Pearson r	0.27066	0.52766	0.1367	-0.03587	-0.09191	0.24724	1	-0.12041	-0.02466	-0.11765	0.05352	-0.03526
	Sig.	0.07213	1.96E-04	0.37056	0.81503	0.54819	0.10154	--	0.43075	0.87226	0.44149	0.72696	0.81811
B. cereus	Pearson r	-0.00141	0.14974	0.02583	-0.2681	0.12367	0.0087	-0.12041	1	0.20693	0.10402	-0.36187	-0.28489
	Sig.	0.99266	0.3262	0.86624	0.07498	0.41831	0.95479	0.43075	--	0.1726	0.49652	0.01458	0.05785
B. flexus	Pearson r	-0.05298	-0.0025	-0.05143	-0.17781	-0.04473	-0.0163	-0.02466	0.20693	1	0.43683	-0.24038	-0.13559
	Sig.	0.72959	0.98699	0.73725	0.24259	0.77048	0.91535	0.87226	0.1726	--	0.0027	0.1117	0.37451
P. fragi	Pearson r	-0.04451	-0.10076	-0.18416	-0.06562	-0.05954	-0.01069	-0.11765	0.10402	0.43683	1	0.0962	-0.11291
	Sig.	0.77157	0.51017	0.22589	0.66845	0.69766	0.94444	0.44149	0.49652	0.0027	--	0.52961	0.46022
P. veronii	Pearson r	0.04837	-0.23991	-0.2124	0.37102	-0.16432	-0.16006	0.05352	-0.36187	-0.24038	0.0962	1	0.31185
	Sig.	0.75237	0.11243	0.16128	0.0121	0.28075	0.29357	0.72696	0.01458	0.1117	0.52961	--	0.03703
P. viridiflava	Pearson r	-0.10346	-0.2362	-0.22509	0.10461	-0.14525	-0.15465	-0.03526	-0.28489	-0.13559	-0.11291	0.31185	1
	Sig.	0.49885	0.11826	0.13711	0.49407	0.34109	0.31042	0.81811	0.05785	0.37451	0.46022	0.03703	--

Static data of relative abundance of each fungal species at the top, middle, and bottom silo positions and different storage time points; analysis of their relationships; Pearson's r represents the correlation coefficient, Sig. indicates the significance level, yellow background indicates a significant correlation at $p < 0.05$ level.
